# Genetic control of iron bioavailability is independent from iron concentration in a diverse winter wheat mapping population

**DOI:** 10.1186/s12870-021-02996-6

**Published:** 2021-05-11

**Authors:** Tally I.C. Wright, Keith A. Gardner, Raymond P. Glahn, Matthew J. Milner

**Affiliations:** 1 NIAB, 93 Lawrence Weaver Road, CB3 0LE Cambridge, UK; 2 Robert W. Holley Center for Agriculture and Health, USDA-ARS, 14853 Ithaca, NY USA

**Keywords:** Iron, bioavailability, Caco-2, MAGIC, wheat, biofortification

## Abstract

**Background:**

Anemia is thought to affect up to 1.6 billion people worldwide. One of the major contributors to low iron (Fe) absorption is a higher proportion of cereals compared to meats and pulse crops in people’s diets. This has now become a problem in both the developed and developing world, as a result of both modern food choice and food availability. Bread wheat accounts for 20 % of the calories consumed by humans and is an important source of protein, vitamins and minerals meaning it could be a major vehicle for bringing more bioavailable Fe into the diet.

**Results:**

To investigate whether breeding for higher concentrations of Fe in wheat grains could help increase Fe absorption, a multiparent advanced generation intercross (MAGIC) population, encompassing more than 80 % of UK wheat polymorphism, was grown over two seasons in the UK. The population was phenotyped for both Fe concentration and Fe bioavailability using an established Caco-2 cell bioassay. It was found that increasing Fe concentrations in the grains was not correlated with higher Fe bioavailability and that the underlying genetic regions controlling grain Fe concentrations do not co-localise with increased Fe absorption. Furthermore, we show that phytate concentrations do not correlate with Fe bioavailability in our wheat population and thus phytate-binding is insufficient to explain the lack of correlation between Fe bioavailability and Fe concentrations in the wheat grain. Finally, we observed no (Fe bioavailability) or low (Fe concentration) correlation between years for these traits, confirming that both are under strong environmental influence.

**Conclusions:**

This suggests that breeders will have to select not only for Fe concentrations directly in grains, but also increased bioavailability. However the use of numerous controls and replicated trials limits the practicality of adoption of screening by Caco-2 cells by many breeders.

**Supplementary Information:**

The online version contains supplementary material available at 10.1186/s12870-021-02996-6.

## Introduction

Iron (Fe) deficiency in humans, also known as anemia, is estimated to effect more than 1.6 billion people worldwide with major implications for many aspects of human health [1]. In places where people’s diet is largely cereal-based, anemia is prevalent, mainly due to the low bioavailability of the Fe in cereals relative to diverse Fe sources such as meat and pulse crops [2, 3]. While much effort has been expended to try and remedy the large number of cases of Fe deficiency globally, anemia is not just a problem in the developing world. It has recently been estimated that more than half of adolescent girls in the UK aged between 11 and 18 years old are also currently anemic [4]. In many developed nations Fe supplementation programs have been in place for several years, for example in the UK a fortification program has been in place since the early fifties. This fortification requirement requires all flours processed in the UK meet the level of Fe > 1.65 mg/100 g of flour. The rationale behind the fortification effort is the high penetration of wheat into an estimated 99 % of households in the UK [5]. Nevertheless, high levels of anemia persist in industrialised nations even with these programs in place [4]. The reason for the failure of these efforts is thought to be down to the form in which Fe is currently added to fortify flours [6]. As most countries use non-recommended, low-bioavailability, atomized, reduced or hydrogen-reduced iron powders to supplement the bread flour [6]. One current strategy for addressing the problem of iron deficiency in humans is through biofortification, with plant breeding or genetic engineering techniques being used to produce new varieties of staple foods with higher iron content in major crops species such as rice, wheat, maize, millet, and legumes. As transgenic foods do not share the same consumer acceptance as traditionally bred varieties, ‘naturally’ bred biofortified crops have been the preferred route to increase Fe intake regardless of GM regulation and public acceptance [7–9].

Substantial natural genetic variation of Fe content of wheat grains has been identified in bread wheat and its progenitors, with Fe levels ranging from 19 to 71 mg/kg [10–12]. This variation is currently being exploited to select iron-rich genotypes for biofortification with the hope to improve iron absorption from the grain [13, 14]. Furthermore, recent attempts to fortify different fractions of the grain suggest that both whole grain and endosperm levels of Fe can be improved both by transgenic means and more traditional breeding [15]. One large problem with this strategy is while Fe concentration in the grain can be improved genetically by breeding for higher Fe levels, this rarely translates to increased Fe absorption by humans [14, 15]. There has been some advancement in the increased concentration and bioavailability of Fe using transgenic means, but it remains to be seen if these varieties will be adopted by a larger consuming public [16–18].

The underlying reason for poor Fe bioavailability from cereals is complex, as the reactive nature of this trace mineral enables strong interactions with other components of the grain that affect Fe bioavailability. Phytate and certain polyphenols and phenolic acids are major components in plant foods that effect Fe absorption [19, 20]. Fifty to sixty % of the iron in cereal grain is bound to inositol hexakisphosphate (IP6) or pentaphosphate (IP5) and forms phytate salts in the aleurone layer of the grain and germ [20, 21]. This is believed to be the main reason for low Fe bioavailability in wheat as the molar excess of phytate complexes the Fe and limits exchange of luminal Fe to the iron transporter, thus preventing absorption in the gut [22, 23]. In addition to phytate, the aleurone layer of grains, is known to contain polyphenols and phenolic acids the majority of which in wheat are phenolic acids, which can both promote and inhibit absorption of Fe by the gut [19, 24]. It has been suggested that wheat may not contain or produce many of these polyphenols and/or that large environmental effects can drastically change the relative amounts of these compounds in the grain and thus might be one of the underlying reasons for the lower reported values of Fe bioavailability in wheat [24–26]. Also more recently different fractions of the grain itself have been shown to inhibit absorption of Fe from grains and can account for a fivefold difference in Fe concentration of the material versus actual Fe absorption [27].

The above factors are the primary reasons why simply increasing Fe intake alone does not always result in more Fe absorption; thus, to properly assess the nutritional quality of Fe in foods, primarily due to the chemical nature of Fe and the degree to which phytochemicals can bind Fe and be present in high molar excess.

While most of the studies presented so far shed light on the mechanisms of bioavailability of Fe from grains, none have investigated the genetic architecture of this complex trait. In order to do so, one must address both Fe concentration and Fe bioavailability together and alone can help increase delivery of Fe, or if bioavailability is a different trait to breed for, is to evaluate both traits in a diverse mapping population and map the quantitative trait loci (QTL) underlying them. Here we use an 8-founder MAGIC (Multiple parent Advanced Generation Inter Crossing) population, encompassing more than 80 % of the genetic polymorphism of UK bread wheat [28], to identify QTL underlying both Fe grain concentration and bioavailability. The population was screened for bioavailability using an established Caco-2 cell bioassay for Fe bioavailability. This model provides a relative measure of absorbable Fe from a given amount of sample, thus providing a practical measure of Fe delivery. In the present study, both Fe concentration and Fe delivery (i.e. bioavailability) were measured from samples collected from two separate field seasons, to identify the stability as well as the possible colocalisation of QTL for both Fe concentration and bioavailability.

## Materials and methods

### Field experiment

Seed were sampled from 1100 MAGIC recombinant inbred lines grown in randomised 1m^2^ nursery plots during the 2015–2016 (“year 1”) and 2016–2017 (“year 2”) field seasons at the NIAB experimental farm in Cambridge, UK, using the agronomy package detailed in Suppl. Table 1. This population was created previously at NIAB and grown in previous field seasons at our Cambridge field site [29]. In year 1, 244 independent BC1F8 offspring lines plus all of the eight founder lines of the MAGIC population were measured for both Fe content and Fe bioavailability. In year 2, 288 independent BC1F9 offspring lines were measured, including all lines from year 1 but only two of the eight founder lines. Twenty grams of seed from each line were dried and milled using a hammer mill (Glen Creston) with a 1 mm sieve for use in later experiments.

### ICP-AES

Fe content of all samples was conducted via inductively coupled plasma emission spectroscopy (ICP-AES). The method used was taken from Glahn et al., 2019 [27]. For each sample 0.5 g flour was dried down and then treated with 3.0 mL of 60:40 HNO_3_ and HClO_4_ mixture in a Pyrex glass tube and left overnight to destroy organic matter. The mixture was then heated to 120 °C for two hours and 0.25 mL of 40 µg/g yttrium added as an internal standard to compensate for any drift during the subsequent inductively coupled plasma atomic emission spectrometer (ICP-AES) analysis. The temperature of the heating block was then raised to 145ºC for two hours. Then, the temperature of the heating block was raised to 190 °C for ten minutes and allowed to cool. The cooled samples in the tubes were then diluted to 20 mL, vortexed and transferred into auto sample tubes to analyze via ICP-AES. The model of the ICP used was a Thermo iCAP 6500 series (Thermo Jarrell Ash Corp., Franklin, MA, USA). Three technical replicates were taken for all lines tested.

### Bioassay for Fe Bioavailability

Whole grain milled wheat flour samples were subjected to simulated gastric and intestinal digestion as per established bioassay conditions [30]. Intestinal digestion is carried out in cylindrical inserts closed on the bottom by a semipermeable membrane and placed in wells containing Caco-2 cell monolayers bathed in culture medium. The upper chamber was formed by fitting the bottom of a Transwell insert ring (Corning) with a 15,000 Da molecular weight cut-off (MWCO) membrane (Spectra/Por 2.1, Spectrum Medical, Gardena, CA). The dialysis membrane was held in place using a silicone ring (Web Seal, Rochester, NY). Iron uptake by the Caco-2 cell monolayers was assessed as previously described and by measuring ferritin concentrations in the cells [30]. The cells were maintained in Dulbecco’s modified Eagle medium plus 1 % antibiotic/antimycotic solution, 25 mmol/L HEPES and 10 % fetal bovine serum. Forty-eight hours prior to the experiment, the growth medium was removed from the culture wells, the cell layer was washed and the growth medium was replaced with minimum essential medium (MEM) at pH 7.0. The MEM was supplemented with 10 mmol/L PIPES, 1 % antibiotic/antimycotic solution, 4 mg/L hydrocortisone, 5 mg/L insulin, 5 µg/L selenium, 34 µg/L triiodothyronine and 20 µg/L epidermal growth factor. This enriched MEM contained less than 80 µg Fe/L. All ingredients and supplements for cell culture media were obtained from GIBCO (Rockville, MD). The cells were used in the Fe uptake experiment at 13 days post-seeding. In these conditions, the amount of cell protein measured in each well was highly consistent between wells. On the experiment day, 1.5 mL of the digested sample was added to the inserts’ upper chamber and incubated for two hours. Then, the inserts were removed and 1 mL of MEM was added. Cell cultures were incubated for 22 h at 37 °C.

The protocols for Caco-2 cell ferritin and cell total protein content analyses were described previously [30]. Briefly, growth medium was removed from the culture well by aspiration and the cells were washed twice with a solution containing 140 mmol/L NaCl, 5 mmol/L KCl and 10 mmol/L PIPES at pH 7.0. The cells were harvested by adding an aliquot of deionized water and placing them in a sonicator (Lab-Line Instruments, Melrose Park, IL). The ferritin and total protein concentrations were determined on an aliquot of the harvested cell suspension with a one-stage sandwich immunoradiometric assay (FER-IRON II Ferritin Assay, Ramco Laboratories, Houston, TX) and a colorimetric assay (Bio-Rad DC Protein Assay, Bio-Rad, Hercules, CA), respectively. Caco-2 cells synthesize ferritin in response to increases in intracellular Fe concentration. Therefore, we used the ratio of ferritin/total protein (expressed as ng ferritin/mg protein) as an index of cellular Fe uptake.

Each plate of samples were run on a 6 well plate with internal controls, consisting of a lentil flour sample, ascorbic acid plus FeCl_2_, FeCl_2_, and MEM media alone. In addition, the eight founder lines were used as overlapping controls across days (1–2 founders per day). For year 2, the founder Claire was run as a control on every plate in addition to the other controls. Finally, three technical replicates of all lines and controls were run on each day.

### Phytate measurements

 Phytic acid was measured using the Megazyme Phytic Acid Assay Kit (Brey, Ireland) according to the manufacturer’s directions. The only change was approximately 100 mg of each flour was digested in 1.8 mL HCl (0.66 M) in 2.2 mL tubes, placed in a rotator mixer with a constant rpm of 20 overnight at room temperature rather than the full 1 g sample suggested. Each sample had three distinct flour samples taken through the whole process to determine phytate amounts for each line tested.

### Data analysis

The grain Fe concentration readings were plotted and visually inspected, there were some extreme outliers present, typically over 80 ppm. High readings were inspected and if a measurement was substantially different to the other technical replicates taken from the same sample or displayed high Al and Ti grain concentration it was removed from the data. These readings were probably soil contaminants. The absolute means were calculated from the three technical replicates of each line and used in the QTL analysis. In year 1, there were four lines with two replicated samples. From these replicates a generalised heritability (*H*^*2*^) was calculated on a line mean basis, implemented by the VHERITABILITY function in Genstat [VSN International. Genstat for Windows, 19th ed.; Hemel Hempstead, UK], which uses an estimation of *H*^*2*^ proposed by Cullis et al. [31]. The replicated samples came from the same field plot, so the *H*^*2*^ calculations provide an estimate of measurement repeatability.

To account for the large day-by-day measurement variation in Fe bioavailability, a lentil flour sample was included as a standardised positive internal control (IC1) on each measurement date in year 1. To improve this adjustment in year 2, two controls were included on each measurement date: a durum wheat flour sample was used as the standardised internal control (IC2) and the MAGIC founder ‘Claire’. Mixed effects linear models using combinations of the different controls as random and fixed factors, and response scaling models where the daily average of the internal control was subtracted from the measurements of corresponding MAGIC lines, were compared in Genstat using Akaike Information Criterion and significance tests of the sample (genotype) effect. Although variance between technical replicates was typically low across both years, in year 2 there were some outliers with high variance between the technical replicates. The individual readings that contributed to the high variance between these technical replicates were removed. In year 2, three genotypes and one internal control were removed which had large variance across all replicates, suggesting possible contamination. The Best Linear Unbiased Predictions (BLUPs) were extracted from the best models and used for subsequent analysis. In year 1, the corrected means of two genotypes with very high ng ferritin/mg protein readings were removed from the dataset as possible contaminants. The same calculations of *H*^*2*^ used for Fe concentration were used for bioavailability. The means of the technical replicates were used in the calculation, all remaining replication came from sampling the same field plots and the *H*^*2*^ estimations were an approximation of measurement repeatability. Cross-trait and cross-year correlations across the population were estimated in R [32] using the Pearson correlation coefficient.

A Shapiro-Wilk test of normality was used to inspect trait distributions and subsequently, QTL mapping was carried out within R using 7367 unique mapped SNP markers from the Illumina Infinium iSelect 90 K SNP wheat array [33] as described in Gardner et al. [29]. Three analysis approaches were used for QTL detection: single marker regression using R/lme4 (IBS, [34]), interval mapping in R/mpMap (IM, [35]) and composite interval mapping with up to three covariates using R/mpMap (CIM). For IBS, two methods of adjustment for multiple-test correction were used. Firstly, a standard multiple-test correction was carried out in R using a False Discovery rate (FDR) correction with a threshold of *p* < 0.05. A second less stringent method used a Bonferroni significance threshold of -log(10) = 3.68, based on α = 0.05/237, where the denominator is the estimated average haplotype number per line in the population, based on map length and number of generations of recombination events. This 2nd method takes into account that markers within haplotypes are highly correlated. For the IM/CIM analyses, an initial liberal cut-off of –log10p < 3 and a window size of 100 markers was used in the mpMap function ‘findqtl’. The mpMap function ‘fit’ was then applied, and QTL retained which had *p* < 0.05 in the fitted model, as well as percentage variation explained > 1 %. In year 1, 237 and 235 MAGIC individuals were used for the QTL analysis of Fe concentration and bioavailability, respectively. In year 2, 284 individuals were used for both traits.

Power analyses were completed with the genotype data of the 235 individuals from year 1, using a custom R script. A single marker was randomly taken as the site of a focal QTL with 100 other markers on other chromosomes used as minor QTL. The power analyses were completed with 4 % variations explained by the focal QTL (5, 10, 25 and 50 %), with the remaining variation shared across the 100 minor QTL. For each percentage variation, 1000 random phenotypes were simulated for each of five heritability values (0.15, 0.25, 0.50, 0.75 and 0.90), achieved by adding random normal variation relative to each heritability. Interval mapping in R/mpMap was then completed with each simulated phenotype, using the same thresholds listed above. A positive detection was recorded when the focal QTL fell within 20 cM of a significant QTL peak.

## Results

### Phenotypic Analysis

#### Fe concentration

**Fig. 1:**
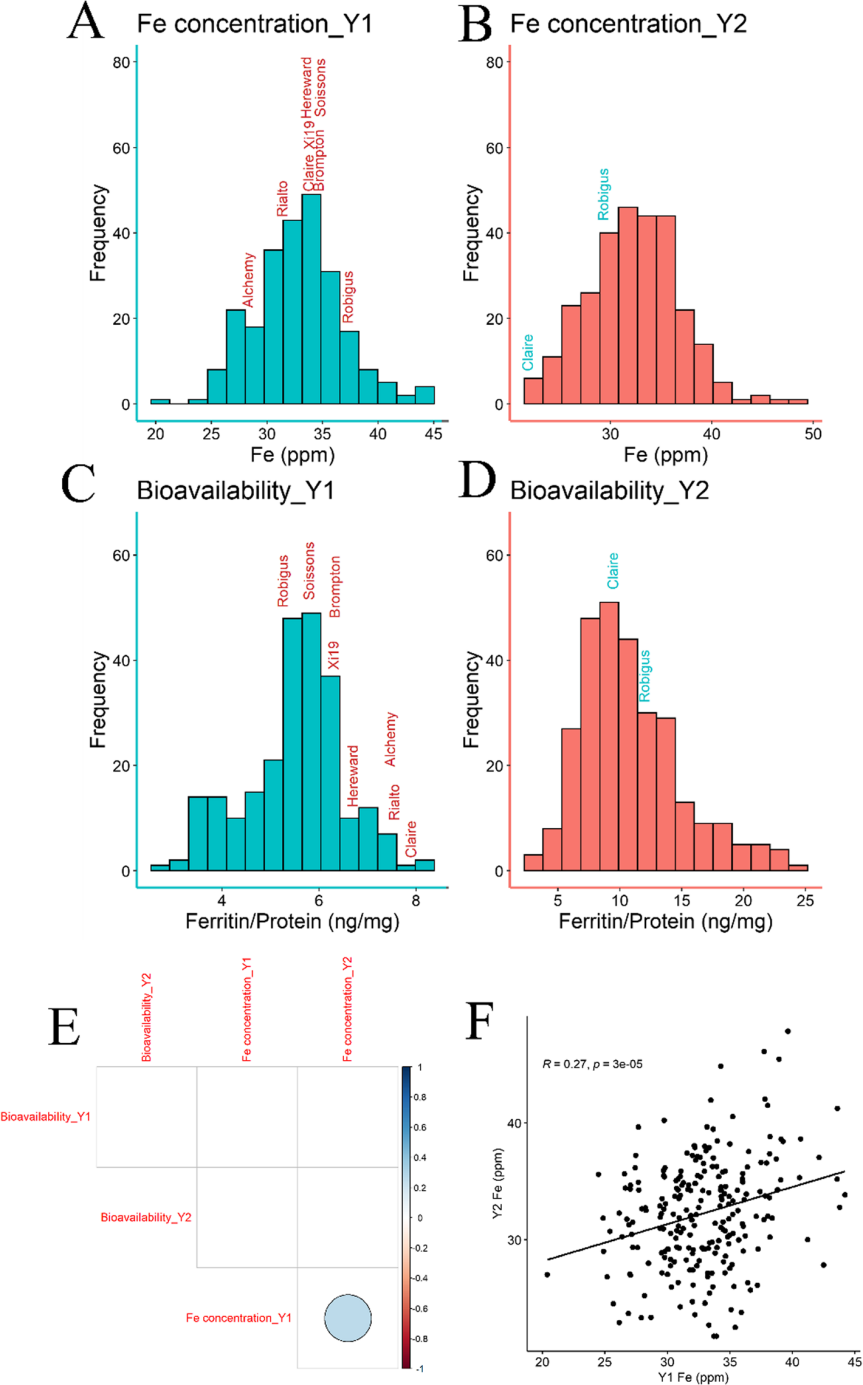
*–* Phenotype frequency plots for the four traits measured. Including the observed means from year 1 and year 2 for grain Fe concentration (**a** and **b**, respectively) and the corrected means (best linear unbiased predictions) for Fe bioavailability in year 1 and year 2 (**c** and **d**, respectively). The MAGIC founder values for each trait are overlaid on each histogram, signified by a text label. In year 1 all founders were measured, while only two founders (Claire and Robigus) were measured in year 2. Also shown is a graphical correlation matrix for the bioavailability line means and the observed Fe concentration means from both years (**e**). Correlations left blank signify that the *P* value associated with the Pearson’s correlation test was greater than *P* = 0.01. The correlation matrix was plotted using the R package “corrplot” [36]. The trend between the Fe concentration means from year 1 (Y1) and year 2 (Y2) is also shown as a scatter plot with the Pearson’s correlation test results overlayed on the plot (**f**)

The absolute means were taken from the three technical replicates and used as the phenotypic data for grain Fe concentration. Observed grain Fe concentrations in the MAGIC population lines ranged from 20.4 to 44.2 ppm in year 1 and 21.7 to 47.9 ppm in year 2. Very similar means were observed across the years (year 1 = 32.8 and year 2 = 32.3 ppm). Once the erroneous measurements had been removed, both distributions appeared to be normal (Fig. 1; year 1: *W* = 0.99, *P* = 0.11; year 2: *W* = 0.99, *P* = 0.15). In year 1, the founder Fe concentrations varied only from 28.2 (Alchemy) to 37.2 ppm (Robigus), suggesting that there was substantial segregation distortion in the population (Fig. 1a). However, one of the two founders measured in year 2, Claire, had an Fe concentration of only 21.7, which fell in the lowest 3 % of the population (Fig. 1b). The other founder measured in year 2, Robigus also showed a considerably different Fe concentration compared to year 1. Furthermore, there was a low *H*^*2*^ observed for grain Fe concentration in year 1 (*H*^*2*^ = 0.19). These trends indicated either the measurement variance of the trait was high, and/or the Genotype x Environment (GxE) interaction was large between years. However, there was a weak but significant correlation between Fe concentrations in year 1 and year 2 across the whole population (r = 0.27, *p* < 0.01, Fig. 1f).

#### Bioavailability

It was observed that there were very large differences between the means of the MAGIC lines for each Caco-2 plate run. To try and normalise for plate variation, a single internal control was included in year 1 and then two controls were included with the year 2 field season samples on each day (Internal control 2 (IC2) and Claire). It would be expected that the controls should follow the same pattern as the line means for each date of measurement, assuming random lines were used each day. In year 1, the line means weakly tracked with the IC1 means. In year 2, line means followed trends in the controls on most days (Suppl. Figure 1) but the IC2 readings were considerably higher than either Claire or the line means.

In year 1, the model which worked best to smooth the large day-by-day measurement variation included the IC1 measurements in the data, with measurement date and genotype treated as random factors. Using this model, *H*^*2*^ was calculated as 0.20 for bioavailability. In year 2 a similar approach was followed: the daily measurements of the controls Claire and IC2 (scaled to have the same mean as Claire) were included in the data, with genotype and measurement date treated as a random factors. In year 2, *H*^*2*^ was higher and estimated as 0.84. It should be noted that ‘heritability’ is only in the context of the experimental set up for measuring bioavailability: all replicated samples in the experiment came from the same field plot. From the two models fitted for each year, the best linear unbiased predictions (BLUPs) were extracted and used in the analysis. The use of two controls in the second year (Claire and the scaled IC2) clearly improved the model.

In year 1, bioavailability ranged from 2.7 to 8.1 ng/mg total protein, with a mean of 5.5 ng ferritin/mg protein (Table 1). Robigus was the founder with the lowest bioavailability (5.2 ng ferritin/mg protein), while Claire showed the highest (7.9 ng ferritin/mg protein), again suggesting the population shows substantial transgressive segregation for this trait. For year 2, bioavailability showed a much greater range from 3.0 to 24.3 with a mean of 10.9 ng ferritin/mg protein, considerably higher than in year 1 (Table 1; Fig. 1d). Furthermore, in year 2 Claire had a marginally different bioavailability of 9.4 ng ferritin/mg protein, whereas there was a substantial increase to 11.9 ng ferritin/mg protein in Robigus. The Shapiro-Wilk test of normality indicated that both distributions were non-normal in distribution (*P* < 0.01); there was a slight left skew in year 1 and a more pronounced right skew in year 2, although we concluded the data skews did not warrant data transformation for QTL mapping. Bioavailability was not significantly correlated between the BLUPs from the different years (Fig. 1e). Furthermore, heritability of models including both years was low. This suggests the inter-annual field environmental variance dwarfs the genotypic effects and/or variance due to measurement error is much larger than genotypic effects. In the latter case, the effect appears to be stronger for the year 1. Furthermore, no significant correlation was detected between bioavailability and iron concentration in either year (Fig. 1e).

**Table 1:** Sample number (*n*), overall trait means (*µ*) and standard deviation (*σ*) for the population individuals used in QTL mapping of Fe concentration and bioavailability. The observed means (Fe concentration) or best linear unbiased predictions (bioavailability) are shown for each of the MAGIC founder lines. For each year, the average standard error of differences between lines was calculated for bioavailability during the model fitting stage (average SED)

Trait	Year	*n*	*µ*	*σ*	Al	Br	Cl	He	Ri	Ro	So	Xi
Bioavailability	1	235	5.5	1.0	7.5	6.3	7.9	6.7	7.5	5.2	5.8	6.3
	*Average SED = 1.49*											
Bioavailability	2	284	10.9	4.1	-	-	9.4	-	-	11.9	-	-
	*Average SED = 1.53*											
Fe concentration	1	237	32.8	4.0	28.2	34.6	33.7	33.5	31.3	37.2	34.7	33.8
Fe concentration	2	284	32.3	4.5	-	-	21.7	-	-	29.2	-	-
Trait units - Bioavailability: Ferritin / Protein (ng / mg). Fe concentration: Fe (ppm)												
*Al* Alchemy, *Br *Brompton, *Cl *Claire, *He *Hereward, *Ri *Rialto, *Ro *Robigus, *So *Soissons, *Xi *Xi19												

### Role of Phytate as an explanation of variation of Fe bioavailability of wheat

**Fig. 2:**
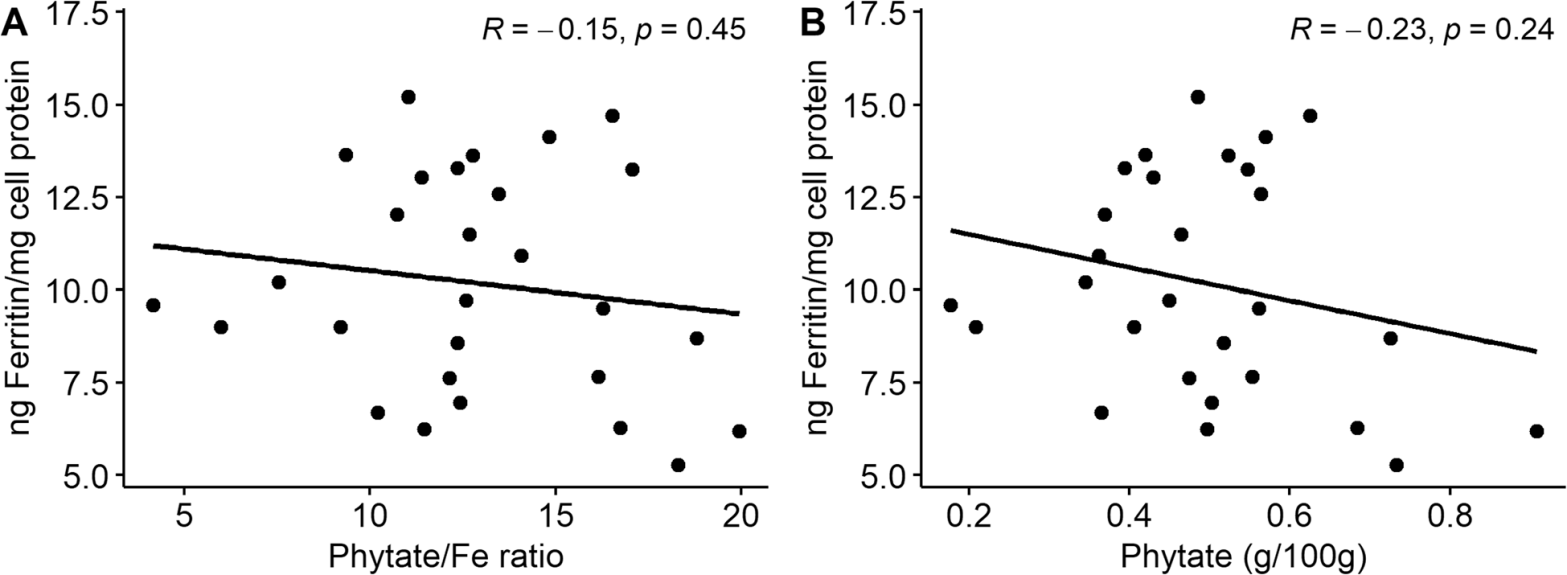
– The role of phytate in explaining variation of Fe absorption. **a** The average ferritin response to whole grain flour from 28 random MAGIC lines grown in year 2 compared to the average ratio of phytic acid to Fe concentrations in the flours. **b** The average ferritin response versus the amount of phytate measured in the milled flour of the same 28 MAGIC lines from year 2

A lot of attention has been paid to the role which phytate plays in Fe absorption. To understand if differences in Fe content and if phytate conentrations in the grain could explain some of the differences seen in the bioavailability between lines, phytate was also measured on 28 MAGIC population individuals from year 2. In the individuals tested, the phytate concentrations varied from 0.18 to 0.91 g of phytate per 100 g flour (Suppl. Table 2). When phytate was shown relative to Fe also present in flour (i.e., the molar ratio of phytate to Fe) there was no significant trend with bioavailability observed (*p* = 0.45, Fig. 2a). There appeared to be a slight negative correlation (R = -0.23) between phytate (g/100 g) and bioavailability. However, this was not significant (*p* = 0.24, Fig. 2b). These results suggest that phytate alone does not explain the differences seen in the bioavailability of Fe of the lines tested.

### QTL mapping

#### Power analysis

The results from the power analyses are shown in Suppl. Figure 2. The probability of finding QTL increased with higher percentage variation explained by the focal QTL and higher heritability associated with the simulated phenotypes. Bioavailability and Fe concentration had an estimated *H*^*2*^ close to 0.2 in year 1. With a trait heritability in this region, the power analyses indicated the probability of finding a QTL explaining 50 % phenotypic variation was less than 25 %, while finding a more minor QTL that explained 5 % variation had a probability of less than 5 %. In year 2, the estimated *H*^*2*^ of bioavailability increased to 0.84. At the higher heritability the probability of finding a QTL that explained 50 % phenotypic variation increased to close to 100 %, while the probability of finding a minor QTL (explaining 5 % variation) was around 20 %. Our heritability estimates in this study were not very precise due to low numbers of reps. Therefore, the true detection power probably lies in between these two extremes.

#### Fe concentration

**Fig. 3:**
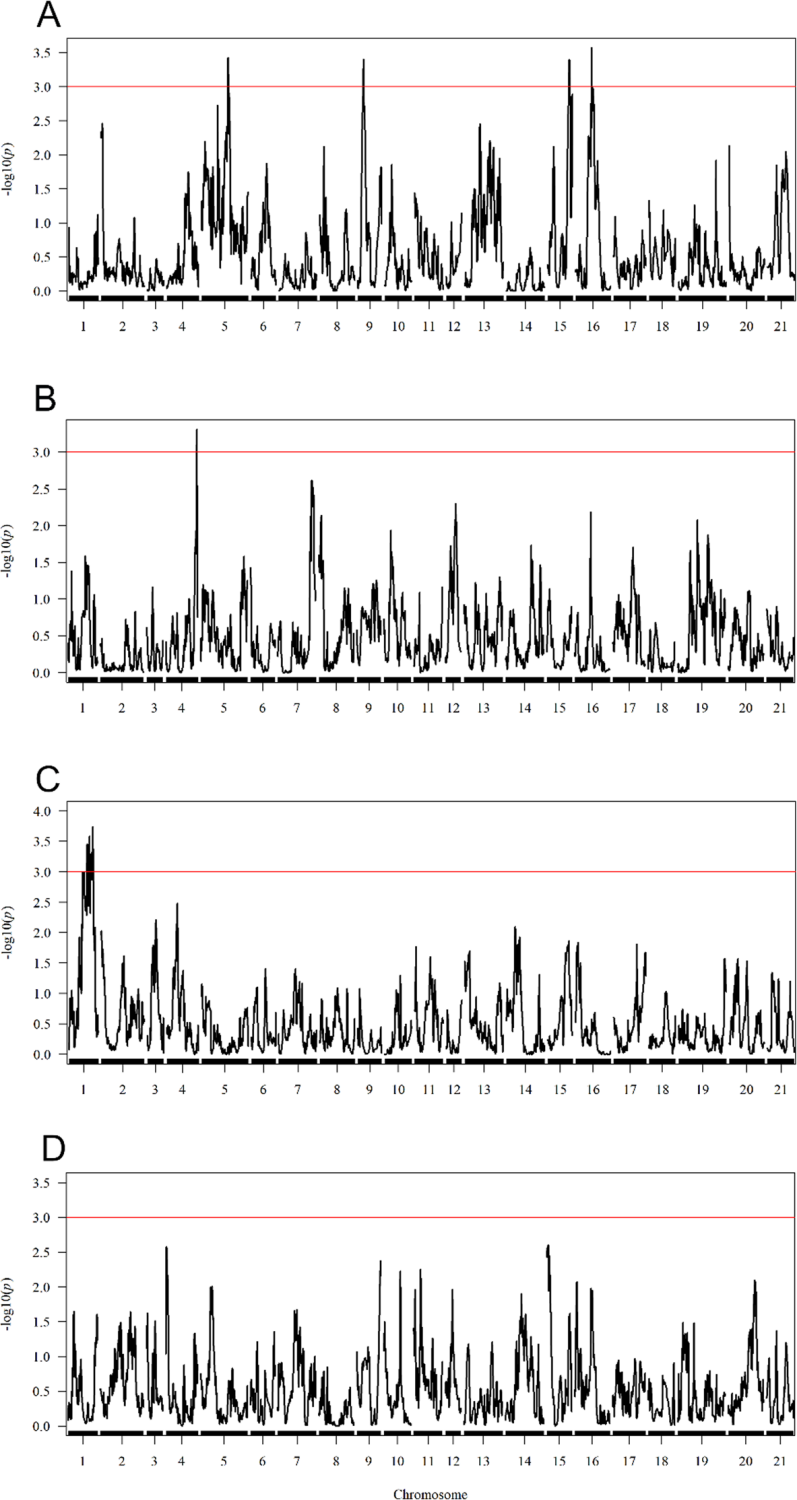
– Interval mapping profiles for the two traits measured across two years. **a** Fe concentration in year (1) **b** Fe concentration in year (2) **c** Bioavailability in year (1) **d** Bioavailability in year (2) For each plot the -log10 (*p*) values are shown across the 21 chromosomes of bread wheat. A -log10(*p*) threshold of 3 is shown as a cut-off for significance. The results show the preliminary output from the interval mapping scan, before the mixed model fitting using ‘fit()’

Across both years, five QTL were identified using IM across five chromosomes (Table 2; Fig. 3), although none co-located between the years. Using CIM, five QTL were mapped to the same chromosomes and an extra QTL was mapped to 29.5 cM on 2B in year 1. The five QTL found through both IM and CIM were approximately mapped to the same location, excluding the QTL on 3D that was mapped to 46.8 cM using IM and 180.1 cM using CIM.

**Table 2:** Candidate QTL for Fe concentration and bioavailability identified through interval (IM) and composite interval mapping (CIM) using mpMap [35]. For each QTL, the table shows the mapped chromosome (Chr) and location (Pos), parental effects with the founder Xi19 used as a baseline, the flanking array markers, the Wald test statistic (Wald) and associated *P* value significance thresholds. The *P* values expressed to –log10 and the percentage phenotypic variation explained by each QTL (% Var) are also included. The results shown were extracted after fitting a multiple QTL model implemented through the mpMap function ‘fit()’

Year	Method	Flanking Markers (left – right)	Chr	Pos (cM)	Al	Br	Cl	He	Ri	Ro	So	Xi	Wald	-log10	% Var
*Fe concentration*															
1	IM	RAC875_c67311_429 – RFL_Contig4718_1323	2B	216.09	-15.7	-33.9	-7.0	-31.8	-14.5	-19.6	-19.3	0	28.08^***^	3.7	7.7
1	CIM	Kukri_c148_1484 – BobWhite_c1149_539	2B	29.5	-0.2	-1.9	-1.1	-0.1	-2.6	-2.0	-2.8	0	20.35^**^	2.3	4.8
1	CIM	RFL_Contig4718_1323 – BS00092235_51	2B	217.5	-3.1	-9.8	-1.6	-19.5	3.3	-8.2	-8.1	0	43.72^***^	6.6	7.4
1	IM	BS00039852_51 – RAC875_c8313_72	3D	46.84	5.0	0.6	-1.5	3.9	-8.9	-2.8	-2.5	0	18.75^**^	2.1	7.7
1	CIM	BobWhite_c42020_456 – Ex_c4296_1270	3D	180.63	-3.2	3.3	1.4	-1.5	2.8	0.2	3.4	0	23.62^**^	2.9	3.9
1	IM	tplb0023j07_1091 – RAC875_c63933_184	5D	175.5	0.0	-0.5	-1.8	1.4	-2.0	-1.8	0.8	0	16.66^*^	1.7	7.7
1	CIM	BS00055493_51 – D_GB5Y7FA02JRQ1I_101	5D	199.08	-1.3	-0.8	-6.5	-0.6	-6.7	-2.8	-1.7	0	37.9^***^	5.5	6.5
1	IM	TA004558_1018 – Ra_c14408_576	6 A	128.93	-3.2	-2.0	-1.1	-2.0	-4.0	-1.4	-0.7	0	22.86^**^	2.7	8.0
1	CIM	TA004558_1018 – Ra_c14408_576	6 A	128.93	-4.5	-3.1	-2.0	-2.4	-5.0	-2.2	-1.8	0	38.43^***^	5.6	8.0
2	IM	BS00012942_51 – Tdurum_contig42013_538	2 A	252.8	-0.4	0.3	-2.9	-2.1	-1.2	-4.0	-4.1	0	26.08^***^	3.3	6.3
2	CIM	BS00012942_51 – Tdurum_contig42013_538	2 A	252.8	-0.6	0.2	-2.9	-2.2	-1.3	-4.0	-4.2	0	26.01^***^	3.3	6.3
*Fe bioavailability*															
1	IM	TA005289_1104 – IAAV3156	1 A	167.31	-0.2	-0.8	0.5	0.5	-0.5	1.2	0.8	0	16.61^*^	1.7	8.1
1	IM	BS00079088_51 – BS00065268_51	1 A	193.67	-0.4	-2.1	1.6	-1.3	-0.1	-0.9	-0.6	0	17.38^*^	1.8	8.4
1	CIM	BS00065268_51 – Kukri_c310_1953	1 A	195	-0.6	-2.8	2.6	-1.1	-0.6	-0.4	-0.2	0	36.65^***^	5.3	8.2
1	CIM	wsnp_Ex_c35331_43499339 – wsnp_JD_rep_c48914_33168544	2 A	87.5	-1.3	0.3	-0.7	-1.1	-2.3	-0.5	-0.6	0	28.89^***^	3.8	5.8
1	CIM	BS00022498_51– RAC875_c1638_165	7B	72.41	0.3	-1.2	-1.7	-0.1	0.0	0.3	-0.1	0	25.29^***^	3.2	3.6
2	CIM	Excalibur_c12980_2392 – wsnp_Ra_c8771_14786376	2 A	10.5	-0.4	3.4	1.8	-0.9	2.5	2.6	1.7	0	24.6^***^	3.0	4.5
2	CIM	BS00084904_51– Excalibur_c100336_106	4B	55.7	-0.5	-2.2	-0.6	-1.4	0.4	-3.0	1.4	0	24.2^**^	3.0	4.4
*Al *Alchemy, *Br* Brompton, *Cl *Claire, *He *Hereward, *Ri *Rialto, *Ro *Robigus, *So *Soissons, *Xi *Xi19															
^***^ = <0.001; ^**^ = <0.01; ^*^ = <0.05.															

For year 1, QTL were found on chromosomes 2B, 3D, 5D and 6 A (Table 2; Fig. 3). The most significant of these hits was present on 2B (217.5 cM). For this QTL, the Xi19 and Rialto haplotypes had the most positive effect and the Brompton and Hereward haplotypes had the most negative effect on Fe concentrations in the grain. Also in year 1, a QTL was mapped to this same region through the IBS method, the peak marker was found at 220.7 cM on 2B with a –log10(*P*) of 3.95 (Table 3). For the other QTL on 2B (29.5 cM) and the single QTL on 6 A, the Xi19 haplotypes also had the most positive effect on Fe concentrations. For the QTL identified on 5D in the CIM approach, Xi19 also contributed the most positive effect, although this was not consistent with the IM approach. The QTL identified on 5D through IM and CIM, was mapped to 175.5 and 199.08 cM, respectively. For these QTL, the Rialto haplotype contributed the most negative effect on the trait, followed by Claire in the CIM, and Claire and Robigus in the IM. Through the IBS method a QTL was also found on 5D at 181.1 cM with a –log10(*P*) value of 4.55 (Table 3), at the peak marker (BS00032035_51) the founders Rialto, Claire and Robigus all shared the same allele, indicating this was the same QTL found in the IM and CIM. For the 3D QTL identified using IM, Rialto also contributed the most negative effect on the trait. However, for the QTL found through CIM on 3D, Rialto contributed a more positive effect indicating that these loci may be different QTL. No percentage variation explained by a QTL was greater than 10 %. The highest percentage variation explained by a QTL was identified was for the QTL mapped to 6 A (8 %) where again the Rialto haplotype contributed the most negative effect on the trait.

**Table 3:** Candidate QTL identified through the IBS mapping. Only QTL with –log10(*P*) values above the Bonferroni significance threshold are shown, which was estimated using population haplotype number. The chromosome the QTL was found on (Chr) and MAGIC genetic linkage map position (Pos) are shown for each QTL hit. The SNP effect represents the fixed effect from each IBS model fitted using lme4 in R [34]. The *P* values were also adjusted using a false discovery rate (FDR) adjustment for total test number

Year	Marker	Chr	Pos (cM)	FDR adjusted *P*	Bonf. threshold	-log10(*P*)	SNP effect
*Fe concentration*							
1	wsnp_Ex_rep_c67543_66165372	2B	220.7	0.1	3.68	3.95	1.14
1	BS00032035_51	5D	181.1	0.06	3.68	4.55	1.11
*Bioavailability*							
1	Ra_c73292_443	5B	91.3	0.21	3.68	3.79	-0.36
2	Excalibur_rep_c110303_320	2 A	18	0.26	3.68	3.71	-1.09

In year 2, no significant QTL were found for the IBS mapping and only a single QTL was found with the IM and CIM approaches: on chromosome 2 A, with the Brompton and Xi19 haplotype having the most positive effect and the Soissons and Robigus haplotype the most negative effect on Fe concentration. This QTL explained 6.3 % of the trait variation. Overall, for the Fe concentration QTL mapped in year 1 and 2, the Xi19 haplotype typically contributed to the most positive effect on the trait, while the Robigus and Rialto haplotypes typically had a negative effect. This pattern was not observed in the phenotypic variation in founders (Fig. 1a and b) where Robgius had the highest Fe concentration across both years, although only two founders were measured in year 2.

#### Fe bioavailability

Fewer QTL were found for bioavailability than Fe concentration. Furthermore, there were no co-located QTL for both traits and the QTL profiles are quite different (Fig. 3) For IM in year 1, two QTL were found on chromosome 1 A with peaks at 167.3 and 193.7 cM (Table 2). The QTL profiles shown in Fig. 3 indicates that these two peaks were linked to the same QTL due to the presence of a long and messy peak along a considerable proportion of 1 A, although it should be noted that the parental effects are not consistent between the QTL (Table 2). However, as there is no significant correlation between the years, the accuracy of determining parental effects might be speculative. The most significant of the IM hits on 1 A was mapped to 194 cM, close to the same hit that appeared through the CIM with a peak at 195 cM. At this locus the Claire haplotype had the largest positive effect on ng ferritin/mg protein and the Brompton haplotype had the most negative effect. There were also two other QTL detected on chromosomes 2 A and 7B through CIM in year 1. These QTL explained a lower percentage of the phenotypic variance than the 1 A QTL (Table 2). An additional QTL was found through IBS mapping on 5B (Table 3), which was not found through IM and CIM.

In year 2, two significant QTL were identified using CIM. One QTL was mapped to 10.5 cM on 2 A and explained 4.5 % of phenotypic variation. At this QTL, the Alchemy and Hereward haplotype contributed the most negative effect on the trait. This QTL was also mapped through the IBS method with a slightly different peak of 18 cM on 2 A (Table 3), at the peak marker (Excalibur_rep_c110303_320) Alchemy and Hereward shared the alternative allele to the other founders. The second QTL found through CIM in year 2 was mapped to 55.7 cM on 4B and explained 4.4 % of phenotypic variation (Table 2). The QTL mapped to 2 A for Fe bioavailability in year 1 is not likely to be the same as the 2 A QTL in year 2. They are 77 cM apart and in year 1, the Rialto founder haplotype contributed the most negative effect on the trait, whereas for the year 2 QTL, Rialto contributed the third most positive effect on the trait. A QTL for Fe concentration in year 2 was also mapped on 2 A, but this QTL was located at the other end of the chromosome (253 cM). Therefore, all three of these QTL are most likely different loci.

## Discussion

Increased Fe concentration has been suggested to be a major breeding target of improved nutrition for humans in crops [37–44]. However, it is important to note that the target of increased Fe concentration assumes that more Fe will be delivered for absorption. Given the chemical nature of Fe and its interaction with phytochemicals such as phytate, phenolic acids and polyphenols, recent studies now show that it is essential to also evaluate the delivery of Fe (i.e. Fe bioavailability) simultaneously with Fe concentration [45, 46].

Thus, our goal was to understand how higher grain Fe concentration in wheat could play a role in increased iron absorption/bioavailability. We measured both Fe content and absorption from two field seasons in > 200 lines of a highly diverse mapping population. A relatively weak (r = 0.27) correlation was observed across years for Fe concentration in the current study. It is possible that this could partially be a result of high measurement variation for the trait. However, we successfully identified four QTL explaining around 30 % of the genetic variation of Fe concentration in total in year 1, and these differed from the single QTL found in year 2, with the QTL profiles between years being noticeably different (Fig. 3). Therefore, we conclude that there is a high level of Genotype x Environment (GxE) interaction for Fe concentrations in the grain, which may have had an impact on the success of breeding for increased Fe concentration [11, 40, 42, 47, 48], despite the evidence of underlying QTL variation in a number of important cereal species [11, 16, 42, 47, 49, 50]. However, it should be noted that the power analyses (Suppl. Figure 2) highlighted that the chances of finding a QTL linked to a phenotype with a *H*^*2*^ of 0.2 was lower than 30 % for all the tested percentage phenotype variations explained by a QTL. For Fe concentration, *H*^*2*^ was estimated as 0.19 in year 1, meaning the probability of finding consistent minor QTL over multiple years was very low. The QTL identified here are different than previous studies in wheat using a biparental population being currently grown in Mexico by CIMMYT, or QTL found in a bread wheat progenitor [13, 49].

The day-to-day variation in the Caco-2 assay for bioavailability presents significant analytical challenges and our year 1 data had insufficient well-distributed controls to accurately estimate trait means across the assays (*H*^*2*^ = 0.20). We were able to improve this in year 2 (*H*^*2*^ = 0.84) but would recommend that more controls (at least three control lines run on every day in addition to the internal control) be used for this system if testing large numbers of samples. This limits the applicability of employing the Caco-2 system for breeding purposes, but it is still far faster than testing on human subjects. There was no correlation between the bioavailability scores across the two years, again suggesting possibly high GxE variation for this trait, although the low accuracy of the year 1 trait mean estimation is likely to have been a significant factor in the lack of a between-year correlation. It is also notable that the range of bioavailability scores within the population was very different between the two years (2.7–8.1, mean 5.5 ng ferritin/mg protein in year 1, 3.0-24.3, mean 10.9 ng ferritin/mg protein in Year 2). Furthermore, the increased variability with higher trait values (e.g. for control IC2 seen in Suppl. Figure 1) suggests that the measurement error may not scale linearly, which would further negatively impact between-year correlation. Nevertheless, a small number of weak QTLs were detected in both years, albeit explaining a relatively low total percentage of the variation. The power analysis in Suppl. Figure 2 showed that there was a good probability of finding a major QTL that explained 50 % of phenotypic variation if the year 2 estimate of *H*^*2*^ for bioavailability was accurate (heritability measured here is only within the ferritin experimental set up). It is possible that the lack of clear major QTL for absorption could be a result of large field fertility effects within trials which were not accounted for here. A larger trial design with biological replication would have been more appropriate for controlling these effects and could have improved our chances of finding consistent minor QTL across years. Given the trait distribution and transgressive segregation (Fig. 1), we think it is most likely that Fe bioavailability is controlled by multiple loci of small effect, most of which were not detectable here. This was supported by the power analysis (Suppl. Figure 2) that showed there was low probability of finding minor QTL (percentage variation explained = 5 %) at either of the different year *H*^*2*^ estimates, which would also explain to why we found no consistent loci across years. For future work with this population, we would recommend increasing the number of MAGIC individuals used which would increase the probability of finding consistent minor QTL across multiple environments. We would also suggest using a larger replicated field design to adjust for field fertility effects. To the best of our knowledge, this is the largest single trial to date to measure and map bioavailability in wheat, and the absence of major QTL in a very diverse population, representing a large percentage of UK polymorphism, provides some insight into why progress in mapping and breeding for bioavailability has been slow. Finally, we note that there was also no correlation between Fe concentration and bioavailability in either year, no QTLs co-locate between the traits, and the QTL profiles (Fig. 3) of the two traits are completely unrelated. This suggests that breeders will have to select not only for Fe concentrations directly in grains, but also increased bioavailability.

The role which phytate plays in Fe absorption is often highlighted in the literature, as nearly 2/3 of the Fe in the grain is thought to be bound to phytate [21]. However, when direct measurements of phytate, Fe concentrations and absorption were all measured on the same samples, phytate concentrations do not explain the variation seen in the ability of Fe to be absorbed by the Caco-2 cells. This suggests that other factors such as polyphenols or other yet identified components may be more important in increasing Fe absorption and not phytate per se. Although the phytate: Fe molar ratio was high for the samples measured which might be why no significant correlation was identified. One major problem with attempting to increase the bioavailability of Fe in wheat is that many of the phenolic acids which have been found to promote the absorption of iron, mainly from beans, do not appear to be produced in wheat [19, 51–53]. It is unknown at this time if wheat cannot make these compounds or if other factors are needed for induction of their production. At the very least, we have not detected any simple explanations for the variation in bioavailability observed here, which is further consistent with it being a complex multi-genic trait.

Finally, as only whole grains were tested in this study, it would be interesting to understand phenotypic variation present in other portions of the grain and if the same QTL can be identified for both increasing Fe concentrations and absorption in the endosperm and germ. Recent studies in maize have shown that the germ itself can be a major inhibitory portion of the grain for Fe absorption and thus fortification of the endosperm which has been done by transgenic means in wheat might be a viable route to increase bioavailable iron [15, 27]. Fe concentrations and phenolic acids are thought to be low in the endosperm, suggesting that the data collected to date will not help increase Fe absorption in white breads [53]. As 70 % of the current consumption of bread is white bread and not wholemeal which would contain the phenolic acids, increasing absorption in this fraction might be more important than in wholegrain/wholemeal bread.

## Conclusions

The large amount of between year variation for both traits and their underlying QTL, the absence of any correlation between Fe concentration and bioavailability, and the lack of major QTL for bioavailability, all highlight why little genetic progress has been made in addressing anemia from cereal based diets. Our results suggest that conventional breeding progress may be best achieved by focusing on iron bioavailability, rather than Fe concentration, and that results will be achieved incrementally via recurrent selection, (enhanced by genomic prediction) rather than by rapid deployment of a small number of major QTL alleles. Otherwise, traditional breeding might not fully address the issue of low Fe absorption in bread wheat.

## Supplementary Information

**Additional file 1:** **Supplementary Figure 1.** A: Bioavailability results from year 1 measured across 13 dates. The daily average of genotypes measured and the internal control 1 (IC1) are plotted. B: Bioavailability results from year 2 measured across 14 dates. The measurements plotted show the daily readings of the internal control 2 (IC2) and Claire and the daily average of the genotypes measured. **Supplementary Figure 2.** Power analysis using simulated phenotypes at 5 heritability values that relate to QTL explaining 4 different percentage variations with 1000 QTL interval mapping runs per simulation. Probability was calculated as the frequency at which the focal QTL was detected in the 1000 QTL mapping runs. 235 MAGIC individuals used in the power analyses, reflecting the number of individuals used in the bioavailability QTL mapping in year 1. **Supplementary Table 1.** Summary of trial inputs and conditions in 2015-16 (year 1) and 2016-17 (year 2). **Supplementary Table 2.** The phytate, bioavailability and Fe levels from year 2 of 28 MAGIC population individuals. Phytate is shown as the mean of three biological replicates (g/100g), bioavailability values are taken from the year 2 corrected means (ng ferritin/ mg of total protein) and Fe is shown as ppm or mg/kg (averaged from three technical replicates).

## Data Availability

Wheat lines used in this publication are freely available upon a signed MTA from https://www.niab.com/research/agricultural-crop-research/resources/niab-magic-population-resources. Please sign and date the MTA, and return it to James Cockram at NIAB, by post or by email as a scanned document. (james.cockram@niab.com)

## Abbreviations

QTL: Quantitative Trait Loci
Fe: Iron
MAGIC: Multi parent Advanced Generation Inter Crossing
IP5: Pentaphosphate
IP6: Hexakisphosphate
ICP-AES: Inductively Coupled Plasma Atomic Emission Spectroscopy
IC: Internal Control
SNP: Single Nucleotide Polymorphism
IBS: Identical By State
IM: Interval Mapping
CIM: Composite Interval Mapping
FDR: False Discovery Rate
BLUP: Best Linear Unbiased Predictions
H^2^: heritability
GxE: Genotype by Environment
MEM: Minimal Essential Media
