# Supplementary material for: Genetic control of iron bioavailability is independent from iron concentration in a diverse winter wheat mapping population
Source: BMC Plant Biol. 2021 May 11;21:212. doi: 10.1186/s12870-021-02996-6 (PMC8112066; doi:10.1186/s12870-021-02996-6)
Supplement: Supplementary file 1 — Additional file 1: Supplementary Figure 1. A: Bioavailability results from year 1 measured across 13 dates. The daily average of genotypes measured and the internal control 1 (IC1) are plotted. B: Bioavailability results from year 2 measured across 14 dates. The measurements plotted show the daily readings of the internal control 2 (IC2) and Claire and the daily average of the genotypes measured. Supplementary Figure 2. Power analysis using simulated phenotypes at 5 heritability values that relate to QTL explaining 4 different percentage variations with 1000 QTL interval mapping runs per simulation. Probability was calculated as the frequency at which the focal QTL was detected in the 1000 QTL mapping runs. 235 MAGIC individuals used in the power analyses, reflecting the number of individuals used in the bioavailability QTL mapping in year 1. Supplementary Table 1. Summary of trial inputs and conditions in 2015-16 (year 1) and 2016-17 (year 2). Supplementary Table 2. The phytate, bioavailability and Fe levels from year 2 of 28 MAGIC population individuals. Phytate is shown as the mean of three biological replicates (g/100g), bioavailability values are taken from the year 2 corrected means (ng ferritin/ mg of total protein) and Fe is shown as ppm or mg/kg (averaged from three technical replicates). [file 12870_2021_2996_MOESM1_ESM.pdf]

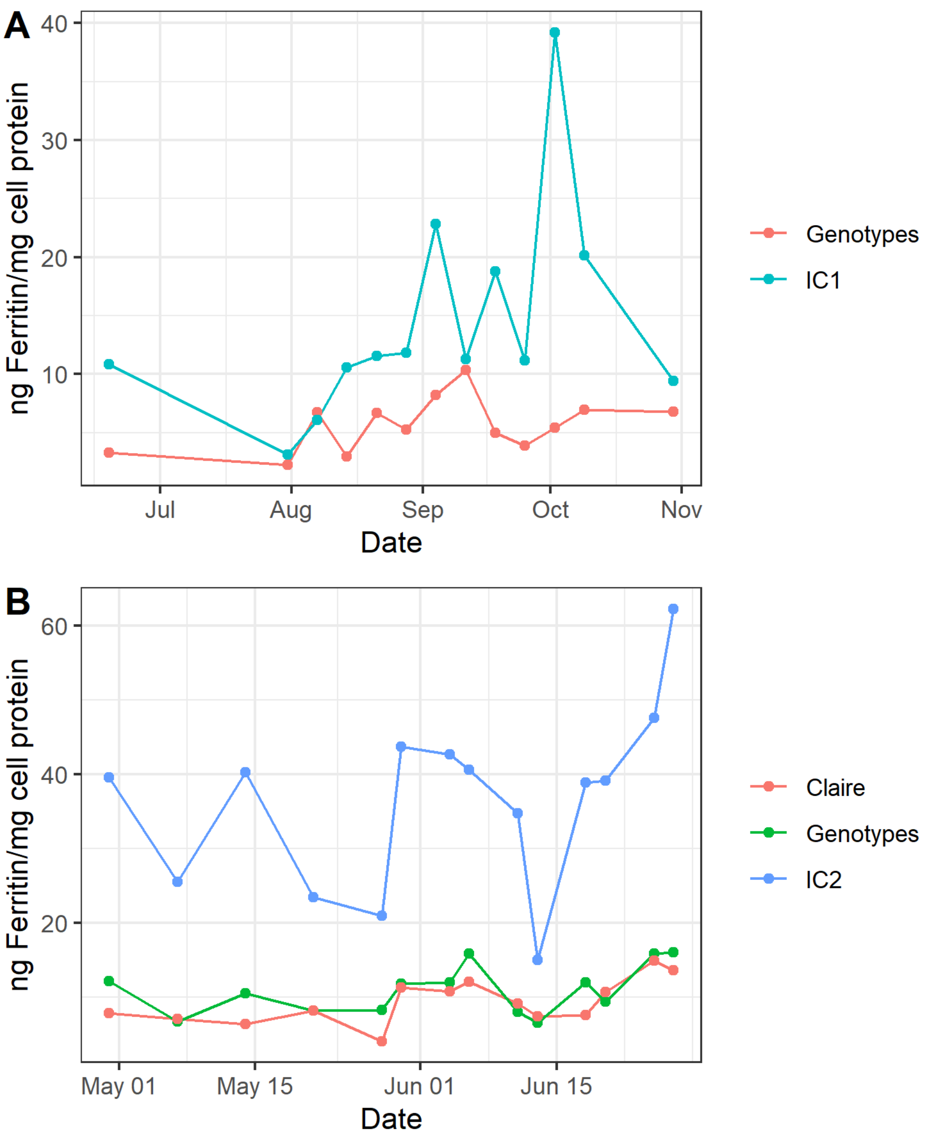

Supplementary Figure 1 – **A**: Bioavailability results from year 1 measured across 13 dates. The daily average of genotypes measured and the internal control 1 (IC1) are plotted. **B**: Bioavailability results from year 2 measured across 14 dates. The measurements plotted show the daily readings of the internal control 2 (IC2) and Claire and the daily average of the genotypes measured.

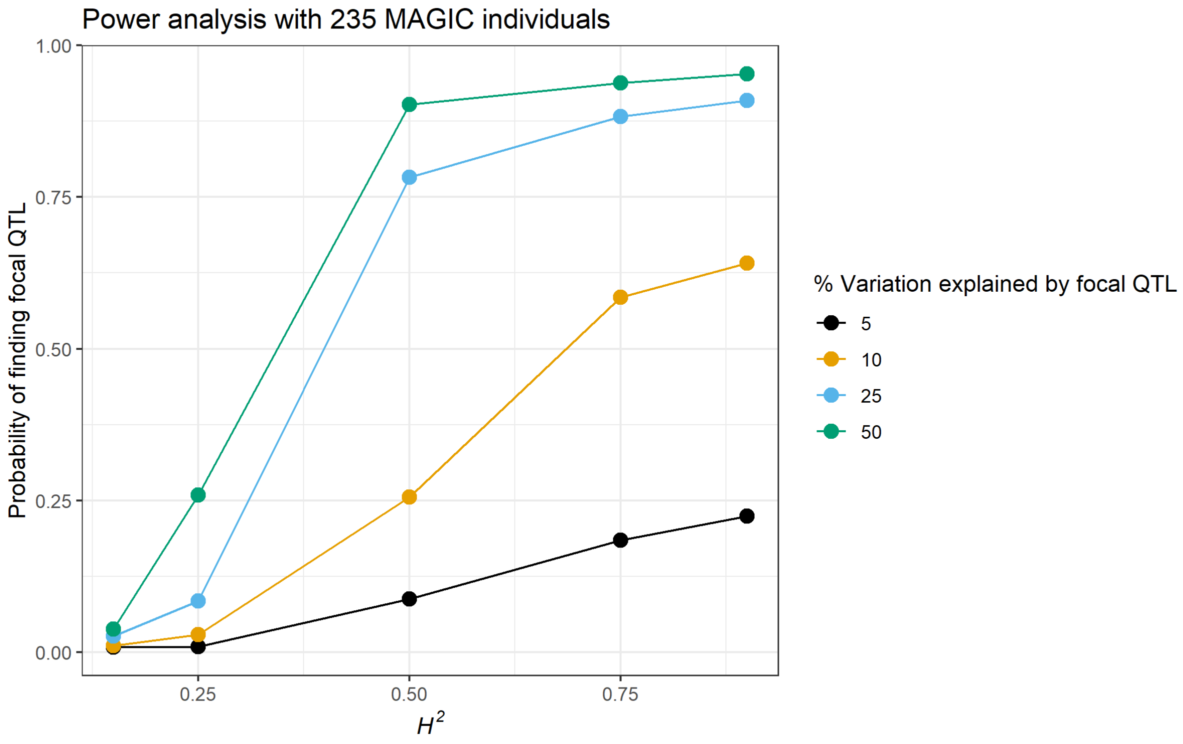

Supplementary Figure 2 – Power analysis using simulated phenotypes at 5 heritability values that relate to QTL explaining 4 different percentage variations with 1000 QTL interval mapping runs per simulation. Probability was calculated as the frequency at which the focal QTL was detected in the 1000 QTL mapping runs. 235 MAGIC individuals used in the power analyses, reflecting the number of individuals used in the bioavailability QTL mapping in year 1.

Supplementary Table 1 – Summary of trial inputs and conditions in 2015-16 (year 1) and 2016-17 (year 2).

**2015-2016**		**2016-17**	
Location (long)	52.23881395	Location (long)	52.24219
Location (lat)	0.095850758	Location (lat)	0.103752
Soil type	Clay	Soil type	Sandy Silt loam
Sowing dates	20/10/2015	Sowing dates	22/10/2016
Harvest dates	16/08/2016	Harvest dates	12/08/2017
Nitrogen fertiliser (kg/ha)	434kg/ha	Nitrogen fertiliser (kg/ha)	434kg/ha
Sulphur fertiliser (kg/ha)	153kg/ha	Sulphur fertiliser (kg/ha)	154kg/ha
**Herbicide applications**		**Herbicide applications**	
18/05/2016	Axial (0.45 lt) wild oats	27/10/2016	Trooper (4 lt) grass and BLWs
18/05/2016	Gal-gone (0.6 lt) BLWs	28/04/2017	Starane XL (1.4 lt) spring herbicide
18/05/2016	Lector (0.1l t) BLWs	28/04/2017	Ally Max SX (35 g) spring herbicide
18/05/2016	Ally Max SX (30 g) BLWs		
**Fungicide applications**		**Fungicide applications**	
13/04/2016	Bravo 500 (1 lt) T0 fung	06/04/2017	Bravo (1 lt) T0 fung
13/04/2016	Tebucur 250 (0.5 lt) T0 fung	06/04/2017	Tebucur (0.5 lt) T0 fung
05/05/2016	Aviator Xpro (1 lt) T1 fung	02/05/2017	Aviator 235 Pro (1 lt) T1 fung
05/05/2016	Joules (1 lt) T1 fung	02/05/2017	Bravo (1 lt) T1 fung
10/06/2016	Proline (0.3 lt) T3 fung	24/05/2017	Adexar (1.5 lt) T2 fung
10/06/2016	Tebucur (0.5 lt) T3 fung	24/05/2017	Chlorthalis (1 lt) T2 fung
10/06/2016	Prosaro (0.05 lt) T3 fung	21/06/2017	Proline 275 (0.45 lt) T3 fung
23/05/2016	Adexar (1.5 lt) T2 fung	21/06/2017	Tebucur (0.45 lt) T3 fung
23/05/2016	Joules (1 lt) T2 fung		
**Insecticide applications**		**Insecticide applications**	
15/03/2016	Karis (0.05 lt) Aphids	N/A	
15/03/2016	Cyren (0.5 lt) WBF		
**Plant growth regulator applications**		**Plant growth regulator applications**	
13/04/2016	Agrovista See 3 (1 lt) 1st PGR	05/04/2017	Agrovista 3 See 750 (1 lt) 1st PGR
13/04/2016	Optimus (0.15 lt) 1st PGR	05/04/2017	Optimus (0.15 lt) 1st PGR
04/05/2016	Agrovista See 3 (1 lt) 2nd PGR	02/05/2017	Agrovista 3 See 750 (1 lt) 2nd PGR

Supplementary Table 2 – MAGIC founder phytate, bioavailability and Fe levels from year 1. Phytate is shown as the mean of three biological replicates (g/100g), bioavailability values are taken from the year 1 corrected means (ng ferritin/ mg of total protein) and Fe is shown as ppm or mg/kg (averaged from three technical replicates).

Founder	Phytate	Bioavail	Fe	mols Fe	mols Phytate	Ratio
Alchemy	1.1	7.5	28.2	0.0000505	0.00161	31.8
Brompton	0.8	6.3	34.6	0.0000620	0.00119	19.3
Claire	1.2	7.9	33.7	0.0000604	0.00187	31.0
Hereward	1.0	6.7	33.5	0.0000600	0.00144	24.1
Rialto	0.8	7.5	31.3	0.0000561	0.00122	21.7
Robigus	1.1	5.2	37.2	0.0000667	0.00173	25.9
Soissons	0.9	5.8	34.7	0.0000622	0.00136	21.9
Xi19	0.9	6.3	33.8	0.0000606	0.00138	22.7

Supplementary Table 2 – The phytate, bioavailability and Fe levels from year 2 of 28 MAGIC population individuals. Phytate is shown as the mean of three biological replicates (g/100g), bioavailability values are taken from the year 2 corrected means (ng ferritin/ mg of total protein) and Fe is shown as ppm or mg/kg (averaged from three technical replicates).

Line	Phytate	Bioavail	Fe	mols Fe	mols Phytate	Ratio
011_4	0.39	13.30	26.94	0.0000483	0.00060	12.36
023_4	0.47	7.63	33.05	0.0000592	0.00072	12.13
025_1b	0.52	8.57	35.38	0.0000634	0.00078	12.36
029_1c	0.68	6.28	34.59	0.0000620	0.00104	16.73
052_3	0.49	15.20	37.18	0.0000666	0.00074	11.04
058_1	0.36	10.91	21.75	0.0000390	0.00055	14.07
078_1bA	0.21	9.00	29.36	0.0000526	0.00032	5.99
081_4	0.18	9.59	35.90	0.0000643	0.00027	4.17
082_2	0.73	5.27	33.86	0.0000607	0.00111	18.30
084_1	0.91	6.19	38.50	0.0000690	0.00138	19.93
097_1	0.42	13.66	37.92	0.0000679	0.00064	9.36
098_1	0.57	14.13	32.50	0.0000582	0.00086	14.83
104_2	0.45	9.72	30.22	0.0000542	0.00068	12.59
104_7	0.46	11.49	30.95	0.0000555	0.00070	12.69
107_1a	0.37	12.03	29.13	0.0000522	0.00056	10.72
113_5	0.55	13.25	27.13	0.0000486	0.00083	17.07
115_1	0.34	10.20	38.67	0.0000693	0.00052	7.54
121_1a	0.55	7.66	28.95	0.0000519	0.00084	16.15
124_2	0.50	6.24	36.60	0.0000656	0.00075	11.47
134B_1a	0.56	9.51	29.18	0.0000523	0.00085	16.27
140_2B	0.73	8.69	32.61	0.0000584	0.00110	18.80
146_1a	0.43	13.03	31.87	0.0000571	0.00065	11.39
160_1	0.56	12.60	35.42	0.0000635	0.00085	13.47
173_2	0.37	6.68	30.22	0.0000542	0.00055	10.21
190_7	0.63	14.71	32.00	0.0000573	0.00095	16.52
199_3	0.41	9.01	37.24	0.0000667	0.00062	9.22
207_1	0.50	6.95	34.23	0.0000613	0.00076	12.43
210_1b	0.52	13.62	34.69	0.0000622	0.00079	12.76
